# Encouragement-Induced Real-World Upper Limb Use after Stroke by a Tracking and Feedback Device: A Study Protocol for a Multi-Center, Assessor-Blinded, Randomized Controlled Trial

**DOI:** 10.3389/fneur.2018.00013

**Published:** 2018-01-25

**Authors:** Jeremia P. O. Held, Andreas R. Luft, Janne M. Veerbeek

**Affiliations:** ^1^Division of Vascular Neurology and Neurorehabilitation, Department of Neurology, University of Zurich and University Hospital Zurich, Zurich, Switzerland; ^2^Cereneo, center for Neurology and Rehabilitation, Vitznau, Switzerland; ^3^Biomedical Signals and Systems, MIRA – Institute for Biomedical Technology and Technical Medicine, University of Twente, Enschede, Netherlands

**Keywords:** stroke, upper limb, rehabilitation, movement sensor, feedback, daily life

## Abstract

**Introduction:**

Retraining the paretic upper limb after stroke should be intense and specific to be effective. Hence, the best training is daily life use, which is often limited by motivation and effort. Tracking and feedback technology have the potential to encourage self-administered, context-specific training of upper limb use in the patients’ home environment. The aim of this study is to investigate post-intervention and long-term effects of a wrist-worn activity tracking device providing multimodal feedback on daily arm use in hemiparetic subjects beyond 3 months post-stroke.

**Methods and analysis:**

A prospective, multi-center, assessor-blinded, Phase 2 randomized controlled trial with a superiority framework. Sixty-two stroke patients will be randomized in two groups with a 1:1 allocation ratio, stratified based on arm paresis severity (Fugl-Meyer Assessment—Upper Extremity subscale <32 and ≥32). The experimental group receives a wrist-worn activity tracking device providing multimodal feedback on daily arm use for 6 weeks. Controls wear an identical device providing no feedback. *Sample size*: 31 participants per group, based on a difference of 0.75±1.00 points on the Motor Activity Log—14 Item Version, Amount of Use subscale (MAL—14 AOU), 80% power, two-sided alpha of 0.05, and a 10% attrition rate. *Outcomes*: primary outcome is the change in patient-reported amount of daily life upper limb use (MAL—14 AOU) from baseline to post-intervention. Secondary outcomes are change in upper limb motor function, upper limb capacity, global disability, patient-reported quality of daily life upper limb use, and quality of life from baseline to post-intervention and 6-week follow-up, as well as compliance, activity counts, and safety.

**Discussion:**

The results of this study will show the possible efficacy of a wrist-worn tracking and feedback device on patient-reported amount of daily life upper limb use.

**Ethics and dissemination:**

The study is approved by the Cantonal Ethics Committees Zurich, and Northwest and Central Switzerland (BASEC-number 2017-00948) and registered in https://clinicaltrials.gov (NCT03294187) before recruitment started. This study will be carried out in compliance with the Declaration of Helsinki, ICH-GCP, ISO 14155:2011, and Swiss legal and regulatory requirements. Dissemination will include submission to a peer-reviewed journal, patient and healthcare professional magazines, and congress presentations.

## Introduction

Although medical interventions and care for stroke patients have been improving tremendously during the last decades, stroke still remains one of the main causes of disease burden globally (1, 2). Frequently reported limitations after stroke are impairments of upper limb motor function and activities, which is present in 80% of the acute stroke patients (3, 4). Recovery of post-stroke impairments mainly occurs within the first 3 months after stroke and plateaus thereafter (5, 6). Although the presence of a plateau phase implies that patients have reached a stable situation, a functional decline has been shown on the long term (i.e., learned-non-use) (7). This underlines the importance of continued practice beyond the first 3 months post-stroke, with the aim to maintain the levels achieved during earlier rehabilitation (8).

Key elements that characterize effective stroke rehabilitation interventions are intensity of practice, and task- and context-specificity (9, 10). Provision of feedback is another important ingredient for effective motor learning after stroke (11). In the last decade, rehabilitation technology has enabled higher intensity of practice and new methods of feedback. Examples of applied technologies for the upper limb are robotics and virtual reality (12, 13). However, these interventions often lack context-specificity, as the training is almost always performed in a clinic and to date, a beneficial effect on what patients actually do with their paretic upper limb in their daily lives has not been reported. Furthermore, many of these technological developments require a high-financial investment in terms of costs and human resources. Recent developments in the field of tracking and feedback technology have provided low-cost tools (e.g., sensors) that incorporate key elements for effective stroke rehabilitation outside the clinic. These tracking and feedback tools have the potential to motivate patients to use their paretic upper limb in daily life while they are in their home environment. With that, they possibly are able to maintain or even improve gains made during the intensive rehabilitation period and facilitate a translation to the patients’ daily life. These tools most often rely on accelerometer data, which have been found to be reliable and valid in stroke subjects (14, 15), and correlate well with patient-reported upper limb use in daily life as assessed by the Motor Activity Log (16). Previous research has shown that stroke survivors have a high adherence to wearing accelerometer devices at home (17) and report good acceptance levels and high usability scores (18, 19). These tracking devices can be used to give augmented feedback regarding movement outcomes or success rate (i.e., knowledge of results) (20, 21). Knowledge of results is an external form of feedback that can encourage a patient to reach a certain goal, such as using the paretic upper limb at least a pre-defined number of times a day (e.g., 100) when performing daily life activities. In this example, the external feedback given by the trackers could enhance a patient’s intrinsic motivation to improve the number of times he or she uses the upper limb during the day. The provided feedback should be tailored to the individual needs to increase motivation, compliance, and effectiveness. There are various feedback modalities available, such as vibrotactile, visual, and auditory feedback. A recent study reported that stroke patients preferred vibrotactile feedback over visual and auditory feedback (19). However, the application of visual feedback on a smartphone has also been indicated as an effective way of delivering feedback (22). With that, the optimal medium for providing feedback remains unknown.

### Study Aim

The primary aim of this study is to determine the post-intervention and long-term effects of wearing a wrist-worn, commercially available tracking device that provides multimodal feedback for 6 weeks on patient-reported amount of paretic upper limb use in daily life in hemiparetic subjects beyond 3 months after stroke, when compared with a control group receiving an identical looking device providing no feedback. The secondary aims are to examine the compliance to use the device, and explore the effect on motor function of the paretic upper limb, upper limb capacity, patient-reported quality of paretic upper limb use in daily life, global disability, health-related quality of life, and during the 6-week intervention, the compliance and activity counts of the paretic and non-paretic upper limb. As a measure of safety, the incidence and severity of side effects related to the long-time wrist-worn feedback intervention during the study period will be investigated. Furthermore, the Minimal Clinical Important Difference (MCID) of the Amount of Use (AOU) subscale of the Motor Activity Log—14 Item Version (MAL—14) will be established.

## Methods and Analysis

### Study Design

The present study is a multi-center, assessor-blinded, Phase 2 randomized controlled trial with a superiority framework, including two parallel study arms. Patients will be informed about the study, including its procedures, and enrolled by a study team member. Randomization will be stratified based on severity of upper limb paresis [Fugl-Meyer Assessment—Upper Extremity subscale (FMA-UE) <32 and ≥32] (23) with a 1:1 allocation ratio. At the time of patient registration in the centralized web-based database (REDCap™), patients will be given a unique study identification number that is linked to a computer-generated randomization assignment using a pre-set list of random numbers. The pre-set list of random numbers was generated based on a seed number (SAS^®^ 9.3) and balanced based on the stratification in blocks of 4. Randomization is performed by a study member not involved in the outcome assessments.

An Emergency Code Break will be available to the investigator. This Code Break should only be opened in emergency situations when the identity of the investigational product must be known by the investigator in order to provide appropriate medical treatment.

This study was approved by the Cantonal Ethics Committee Zurich and Cantonal Ethics Committee Northwest and Central Switzerland (BASEC-number 2017-00948). All subjects will give written informed consent in accordance with the Declaration of Helsinki. The trial is registered at https://clinicaltrials.gov, unique identifier NCT03294187 prior to patient recruitment.

### Eligibility Criteria/Participants

This study includes subjects aged 18 years or older who have experienced a unilateral stroke more than 90 days ago and have residual upper limb paresis after completion of all inpatient rehabilitation. Subjects have to be able to lift the paretic arm against gravity (>30° of flexion or abduction), don/doff the devices on both wrists independently or with the assistance of a caregiver, and provide informed consent as documented by signature. Patients will be excluded from this study if they have major untreated depression, severe cognitive impairment, comprehensive aphasia, and/or severely impaired sensation (unable to feel a soft touch on the dorsal side of their paretic wrist with closed eyes). Other exclusion criteria are a potential non-compliance such as hospitalization during the study period, known intolerance to the device material, known drug or alcohol abuse, and/or other major comorbidities (e.g., cardiopulmonary disease, renal failure, hepatic dysfunction, and orthopedic disorders of the upper limb).

### Stepwise Procedures

**Figure 1:**
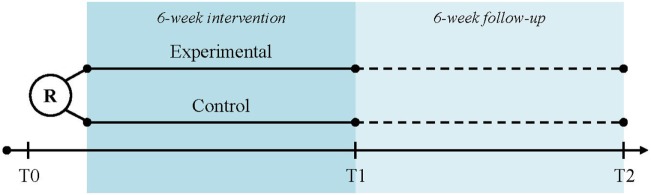
Participant time line. Legend: R, randomization; T0, baseline assessment; T1, post-intervention assessment; T2, follow-up assessment.

Subjects will be recruited at three study centers in Switzerland: University Hospital Zurich (academic hospital), cereneo—Center for Neurology and Rehabilitation (rehabilitation clinic), and Zürcher RehaZentrum Wald (rehabilitation clinic) by the local principal or sub-investigator. The participant timeline is displayed in Figure 1. The written participant information and informed consent forms (in German) can be obtained by the corresponding author. After having obtained participant consent, the baseline assessment (T0) will be performed. Randomization will take place after the baseline assessment and depending on the group allocation, participants will be handed over the trial arm-specific devices and receive instructions accordingly. Randomization and device hand-over will be performed by study personnel not involved in assessments (e.g., physical therapist and study nurse). The intervention period has a total duration of 6 weeks. The post-intervention assessment (T1) takes place at day 45 ± 3 after baseline and the follow-up assessment (T2) at day 90 ± 6 after baseline.

Recruitment started in September 2017 and is expected to last until December 2018, which means that the estimated study completion will be in March 2019. If inclusion stays behind, ethical approval will be obtained for adding other study centers in order to enroll the required number of patients within this timeframe. No payment will be provided to the participants other than compensation for travel costs.

### Interventions

**Figure 2:**
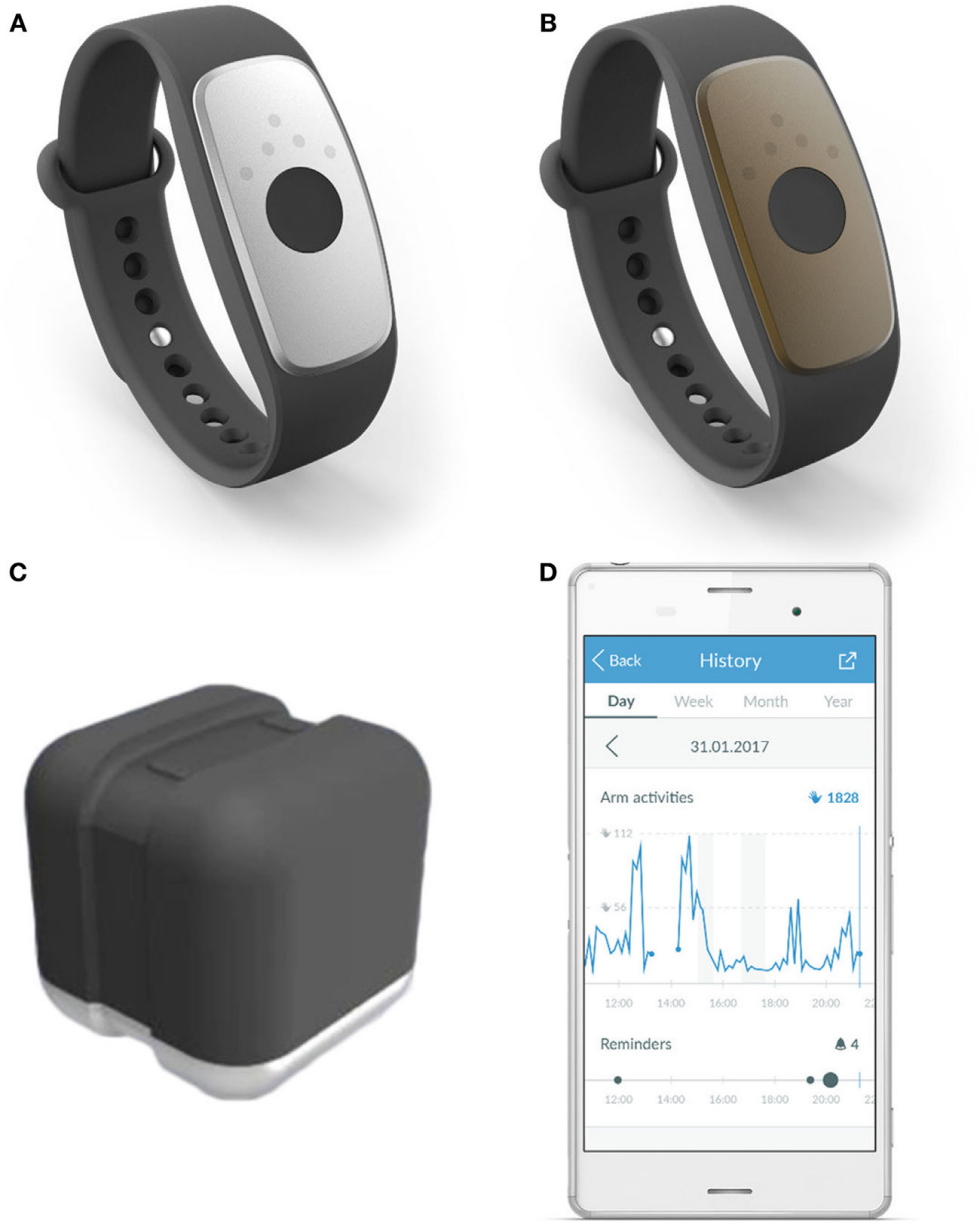
Investigational devices. Legend: **(A)** motion tracker “ARYS™ me|tracker”, black-silver tracker; **(B)** “ARYS™ pro|tracker healthy”, black-brown tracker; **(C)** accessory charging station “ARYS™|tracker charger”; **(D)** android smartphone with the pre-installed application “ARYS™ me|app,” here displaying the history of today’s Arm Activities, including provided reminders.

The investigational devices to be used in the study are (see Figure 2):

- motion tracker “ARYS™ me|tracker” (Figure 2A: black-silver tracker); and
- motion tracker “ARYS™ pro|tracker healthy” (Figure 2B: black-brown tracker).

Additionally, the patients receive:

- two accessory charging stations “ARYS™|tracker charger” (Figure 2C);
- an Android smartphone with the pre-installed Application “ARYS™ me|app”; only for study subjects in the intervention group (Figure 2D); and
- a manual covering the following topics: study system components, charging the trackers, placement of the trackers, when not to use the trackers (e.g., bathing, swimming, MRI scanning, and uncomfortable feeling on the wrist), Frequently Asked Questions regarding technical problems and cleaning, and contact information. Additionally, the manual for the experimental group provides information regarding on-device feedback and reminders, use of the smartphone app, and daily goals.

The devices are manufactured and distributed by “yband therapy AG” and are intended for use in arm therapy of patients with arm movement deficiencies. The arm movement deficiencies may have been caused by cerebral, neurogenic, and spinal-related disorders. The “ARYS™ me|tracker” and “ARYS™ pro|tracker healthy” are both CE-certified as a “Class 1 Medical Device.”

#### Experimental Intervention

Participants in the experimental group will wear an “ARYS™ me|tracker” on the paretic wrist and an “ARYS™ pro|tracker healthy” on the non-paretic wrist (see Figure 2). The trackers are hardware-wise identical and consist of: the actual tracker (silver with a feedback and communication module for the paretic side, which is deactivated for the control group); a reference tracker (black-brown tracker for the non-paretic side); and an exchangeable, flexible black wristband. Both elements are made out of biocompatible materials. The devices should be worn every day, as long as possible over a period of 6 weeks. During this 6-week period, a three-axis accelerometer in each device will constantly monitor the subjects’ arm movements. A threshold of 0.1 g acceleration is defined for detection of movements. This raw acceleration data, aggregated over 1 min, will be converted on-device in so-called “Arm Activities.” Starting from a pre-defined goal-value, new daily goals will be automatically calculated every day from the rolling average of the last 30 days plus 3% (e.g., after the first intervention week, patient X shows an average number of 100 “Arm Activities” per day. For day 8, his daily goal therefore becomes 103 “Arm Activities”). Patients will constantly be challenged to slightly increase their Arm Activities without ever being overstrained. Assuming a steady arm activity during the course of the day, a linear target activity line will be calculated (default start/end times: 8:00 a.m. to 10:00 p.m.). The already reached “Arm Activities” will be compared with an intermediate target value. After 30 min of inactivity, the “ARYS™ me|tracker” vibration engine will provide the subject on the paretic wrist with two short double pulses and up to four LEDs will light up in orange color to remind to make more use of this arm (Level 1 Reminder). If 30 min later, “Arm Activities” are still below the intermediate target, a second escalation level of the reminder is triggered: five long vibrating pulses will be provided and up till four LEDs will light up in red color (Level 2 Reminder). Thereafter, the escalation level is reset and starts over again with the Level 1 reminder next time. Additionally, by pressing the button on the “ARYS™ me|tracker” once, the LED lights will show the percentage of the daily target that is already reached (one white LED = <25%; two white LEDs = 25–49%; three white LEDs = 50–75%; three white LEDs and one blinking LED = 75–99%; four white LEDs = 100%, daily target reached) and the patient receives vibration feedback. When the patient has reached the daily target, the tracker vibrates.

**Figure 3:**
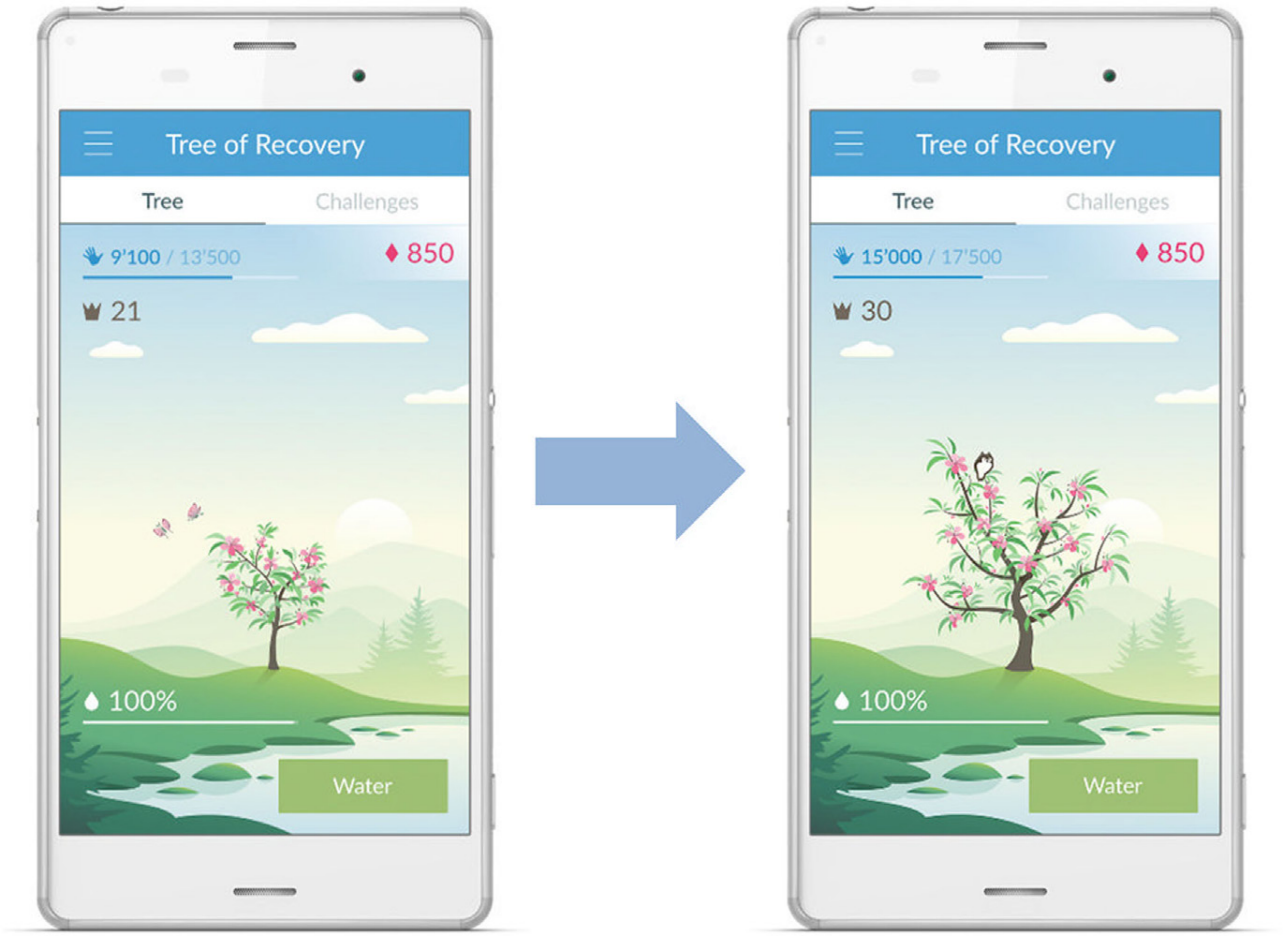
“Tree of Recovery.” Legend: a figurative representation of the amount of upper limb use in daily life. Arm Activities are displayed in blue numbers and the earned diamonds in red.

Participants in the intervention group are encouraged to regularly check the pre-installed “ARYS™ me|app” on a standard commercial Android smartphone, which they have received. It will download “Arm Activity” data from the “ARYS™ me|tracker” automatically, motivate study subjects based on the concepts of gamification, and visualize activity data over various time intervals. Patients can monitor the development of their daily targets, today’s progress, and provided reminders toward the daily target, as well as a history of all past “Arm Activities” and reminders presented in a day, week, month, and year view (see Figure 2D). Additionally, they can follow the growth of the “Tree of Recovery” (see Figure 3). This is a figurative representation of their amount of upper limb use over time, in which they earn “Diamonds” by fulfilling challenges of intensive activity and can use these “Diamonds” to water their tree to let it grow and flourish.

For using the devices, no medical and/or surgical procedures will be involved. However, a tight fit on the wrist will be beneficial to reduce motion artifacts and to ensure that the vibration feedback is properly felt by the study subjects. As both devices are splash-waterproof, there is no need to doff the trackers during daily activities involving water, such as washing hands or showering. Thanks to the easy-to-use pin-and-tuck closure, study subjects should be able to don/doff the device on their own without any help (see Figures 2A,B). Other than instruction through a non-blinded study member at one of the participating study sites and the information provided in the group-specific leaflet, no training or experience will be required to use the devices. The one-to-one instruction takes up to 30 minutes. Furthermore, the patients can always look up information in the accompanying manual, pose questions during the weekly telephone calls, and call the hotline.

#### Control Intervention

Subjects in the control group will use the same devices in the same way as the intervention group. They will wear the “ARYS™ me|tracker” on their paretic and the “ARYS™ pro|tracker healthy” on their non-paretic wrist. The “ARYS™ me|tracker” will have a custom firmware installed that deactivates both the vibration module as well as the LED-progress bar. Therefore, subjects in the control group will neither receive nor know about any feedback happening in the intervention group. They will not receive a smartphone with the “ARYS™ me|app.” The one-to-one instruction for the control intervention takes up to 15 minutes. Analogue to the patients in the experimental group, they can consult the accompanying manual, ask questions during the weekly telephone calls, and, when needed, contact the hotline.

#### Additional Information Regarding the Interventions

To increase study compliance and adherence, both intervention and control group subjects will receive weekly reminder calls (six in total) to wear the “ARYS™|tracker” devices every day. These calls will be performed by an unblinded study team member. Additionally, there will be a hotline telephone number they can call when facing any technical problems. Discontinuation of the allocated intervention for a given trial participant will take place in case of, for example, occurrence of a Serious Adverse Event (AE) or participant request. We expect no modifications on the given intervention during the study. All participants are permitted to receive concomitant rehabilitation interventions such as physical therapy and/or occupational therapy during the trial. The time spent in these interventions will be registered during the total study period.

### Sample Size

A sample size calculation based on a difference between groups in change from baseline to post-intervention in the AOU subscale of the MAL—14 of 0.75 points. With an effect SD of 1.00, a two-sided alpha level set at 0.05, and a power of 80%, 28 subjects per group are required (24). Taking into account an attrition rate of 10%, a total of 62 subjects will be randomized. In case of an attrition rate higher than the expected 10%, post-recruitment for replacement will take place.

### Measures

Potential participants will be screened and examined by the local study team for eligibility. Included subjects will be assessed prior to randomization (T0), after the 6-week intervention (T1), and 6 weeks thereafter (T2). The outcome assessors will be trained regarding the assessments and are blinded to group allocation. Study participants will be told at the beginning of the study, as well as right before and during the T1 and T2 assessments, not to mention any details regarding their experiences with the devices during the assessment visits. Potential success or failure of blinding will be checked, by asking the assessors at the end of the post-intervention and follow-up visits, in which group they think a given patient was allocated. Success of blinding will be presented in a descriptive manner.

Primary endpoint is the change from T0 to T1. An overview of the measures and timing of assessments is displayed in Table 1. Data collection forms (in German) can be obtained by the corresponding author upon request.

**Table 1:** Overview of study measures.

Measure	Domain measured	T0	I	T1	T2
**Primary outcome**					
Motor Activity Log—14 Item Version (MAL—14) (Amount of Use)	Patient-reported amount of upper limb use in daily life	X		X	X

**Secondary outcomes**					
MAL—14 (Quality of Movement)	Patient-reported quality of upper limb use in daily life	X		X	X
Fugl-Meyer Assessment—Upper Extremity	Upper limb motor function	X		X	X
Action Research Arm Test	Upper limb capacity	X		X	X
EuroQol Five Dimensions Five Levels Questionnaire	Health-related quality of life	X		X	X
Modified Rankin Scale	Global disability	X		X	X
Global Rating of Perceived Changes	Self-perceived change			X	X
Quantitative Sensor Data	Compliance; activity counts of the paretic and non-paretic side		X		
Adverse Events	Safety	X	X	X	X

**Other measures**					
Demographics	Participant demography	X			
Stroke Event Data	Disease characteristics	X			
Charlson Comorbidity Index	Medical history	X			
National Institutes of Health Stroke Scale	Neurological impairments	X			
Edinburgh Handedness Inventory	Handedness	X			
Apples Test	Visuospatial neglect	X			
Concomitant Therapy	Standard rehabilitation therapy	X		X	X

*I, intervention; T0, baseline; T1, post-intervention; T2, 6-week follow-up*.

#### Primary Outcome

The primary outcome measure of the present study is the patient-reported amount of use of the paretic upper limb in daily life, measured with the MAL—14 AOU subscale (24–26). In a semi-structured interview, the patient is questioned regarding how often he or she has used the paretic upper limb during 14 activities of daily living (ADL) tasks on a six-point ordinal scale. For each item, the score ranges from 0 [did not use my weaker arm (not used)] to 5 [used my weaker arm as often as before the stroke (same as pre-stroke)]. Scores are only given if the patient has performed the ADL task during the last week. The total score for the MAL—14 AOU subscale ranges from 0 to 5, calculated by adding scores for each of the performed items and subsequently dividing this number by the total number of performed tasks.

#### Secondary Outcomes

The secondary outcome measures include the FMA—UE, Action Research Arm Test (ARAT), EuroQol Five Dimensions Five Levels Questionnaire (EQ-5D-5L), modified Rankin Scale (mRS), MAL—14 Quality of Movement (QOM) subscale, Global Rating of Perceived Changes (GRPC), Quantitative Sensor Data for compliance and Arm Activities of the paretic and non-paretic upper limb during the 6-week intervention, and AEs.

##### Fugl-Meyer Assessment—Upper Extremity Subscale

The FMA-UE measures motor function of the paretic upper limb (27, 28). A total of 33 items are assessed and each item is rated on a three-point ordinal scale (0 = cannot perform, 1 = performs partially, and 2 = performs fully). The sum score ranges from 0 to 66.

##### Action Research Arm Test

Upper limb capacity is measured with the ARAT and includes four categories: grasp (six items), grip (four items), pinch (six items), and gross movement (three items) (29–31). Each item is rated on a four-point ordinal scale (0 = no movement; 1 = the movement task is partially performed; 2 = the movement task is completed, but takes abnormally long; and 3 = the movement is performed normally), leading to a maximum overall score of 57 points.

##### EuroQol Five Dimensions Five Levels Questionnaire

The EQ-5D-5L is a self-completed questionnaire regarding health-related quality of life (32, 33). Each of the five assessed domains (mobility, self-care, usual activities, pain/discomfort, and anxiety/depression) is described by one out of five responses, ranging from “no problem/not at all” to “unable/major problem.” In addition, the patient is asked to rate his or her self-rated health on a Visual Analog Scale. Approval of the EuroQol Research Foundation to use the German (Switzerland) EQ-5D-5L paper version has been obtained (March 13, 2017).

##### Modified Rankin Scale

Global disability is assessed with the mRS, which includes an informal interview with the patient, to rate the extent of a patient’s post-stroke disability or impairment in ADLs on a six-point ordinal scale, ranging from 0 = “no symptoms at all” to 5 = “severe disability” (34–36).

##### Motor Activity Log—14 Item Version—Quality of Movement Subscale

The MAL—14 QOM measures the patient-reported quality of paretic upper limb use in daily life by performing a semi-structured interview (24–26). Analogue to the AOU subscale, 14 ADL tasks are questioned. Patients score how well the paretic upper limb helped during this activity on a six-point ordinal scale, with item scores ranging from 0 [my weaker arm was not used at all for that activity (not used)] to 5 [the ability to use my weaker arm for that activity was as good as before the stroke (normal)], and a total score ranging from 0 to 5.

##### Global Rating of Perceived Changes

With the GRPC, the patients rate their perceived changes in the daily life usage of the paretic upper limb, using the following seven-point Likert scale: score 1 is much better; score 2 = a little better, meaningful; score 3 = a little better, not meaningful; score 4 = about the same; score 5 = a little worse, not meaningful; score 6 = a little worse, meaningful; and score 7 = much worse (37).

##### Quantitative Sensor Data for Compliance and Activity Counts

Adherence will be monitored through the weekly reminder calls and through analysis of the gathered Quantitative Sensor Data and smartphone App usage data. The Quantitative Sensor Data measure the “Arm Activities” by using the acceleration data, aggregated over 1 min during wearing the activity tracker. This refers to Arm Activities of both the paretic and non-paretic side and allows comparing both changes in Arm Activities over time for each upper limb during the 6-week intervention, as well as comparing Arm Activities between the paretic and non-paretic upper limb.

##### Safety

Adverse Events will be gathered from inclusion upon follow-up. Assessments will be made during each study visit, as well as by the weekly phone calls during the intervention period. Each time, study subjects will be actively questioned by unblinded study personnel whether they experienced any AEs. In case of occurrence of a related AE, the time of onset, the duration, the resolution, actions that were taken, the intensity as well as the relationship with the study intervention will be recorded.

#### Descriptive Measures

Next to the above described outcome measures, the following data will be collected at baseline to characterize our cohort: demographic data, stroke event data, comorbidities, handedness [Edinburgh Handedness Inventory (38)], neglect [Apples Test (39)], social situation, and neurological functions [National Institutes of Health Stroke Scale (40)].

### Data Collection

Assessments will take place at one of the participating centers, or at the patients’ home when he or she is not able to visit one of the study centers. All assessors will be trained prior to the first screening during which they will receive proper instruction and guidance regarding all outcome parameters and assessments that will be taken. The trial management committee (TMC) will be available for questions.

In the case that a patient discontinues or deviates from the intervention protocol, all efforts will be made to obtain at least the primary outcome measure (MAL—14 AOU) and the safety measures (AEs) at the pre-defined study visits. All patients will receive a reminder for each visit to diminish retention and incomplete follow-up.

### Data Management

Study data will be recorded in electronic Case Report Forms (eCRF). For each enrolled study participant, an eCRF will be maintained. These eCRFs will be kept current to reflect subject status at each phase during the course of study. The participants’ code will be assigned in a random order. Source data will be available at the respective study site to document the existence of the study participants. Source data will include the original documents relating to the study, as well as the medical treatment and medical history of the participant. All study data will be archived for a minimum of 10 years after study termination or pre-mature termination of the clinical trial in a secure database. Study data will be managed using REDCap™, being hosted on servers administered by the Data Informatics Services Core of cereneo Schweiz AG. To access the REDCap™ site, all study team users of the REDCap™ system will be issued a unique username and password that is generated and maintained by our administrator on the REDCap™ server.

The study data will be analyzed by the TMC after study completion (last patient, last visit). No interim analysis will be performed. Only the study coordinators and principle investigators will have access to the final trial dataset.

### Statistical Analysis

Patient demographics and baseline data will be analyzed by trial arm using summary descriptive statistics. Baseline differences between groups will be tested by the independent-samples *t*-test for parametric data, Pearson’s χ^2^ test for categorical data, and the Mann–Whitney *U* test for non-parametric data.

Differences in change scores (T0–T1) between groups will be analyzed for the primary outcome (MAL—14 AOU) and secondary outcomes (MAL—14 QOM, FMA—UE, ARAT, EQ-5D-5L, mRS, and Quantitative Sensor Data). In addition, change will be monitored in the follow-up assessments (T1–T2). An analysis of covariance will be applied, with the baseline value of the measure of interest as a covariate. Compliance to using the devices during the 6-week intervention period and safety data will be presented by trial arm with descriptive statistics and compared between groups by the independent-samples *t*-test, Pearson’s χ^2^ test, or Mann–Whitney *U* test, depending on the nature of the data distribution. In a secondary analysis, compliance to wearing the trackers—based on the Quantitative Sensor Data—will be added as a covariate for analysis of the primary outcome MAL-14 AOU.

All randomized subjects will be included in the analyses according to the intention-to-treat approach. Missing data will be imputed. In addition, differences in baseline characteristics of patients who did and did not dropout will be formally tested. A *p*-value of <0.05 will be considered to be statistically significant. The analyses will be performed in IBM SPSS Statistics 23.

Furthermore, the compliance to the intervention will be presented with descriptive statistics. The MCID will be calculated based on the MAL—14 AOU and GRPC scores, analogue to previous work by Lang et al. (37).

### Monitoring and Quality Control

The sponsor–investigator has the overall responsibility for the study conduct. The TMC at the University Hospital Zurich provides day-to-day support for the sites.

Regular monitoring visits at the investigator’s site prior to the start and during the course of the study will help to follow-up the progress of the clinical study, to assure utmost accuracy of the data, and to detect possible errors at an early time point. All original data including patient files, progress notes, and copies of laboratory and medical test results will be available for monitoring. The monitor will review all or a part of the eCRFs and written informed consent forms. The accuracy of the data will be verified by reviewing the above referenced documents. A formal Data Monitoring Committee or equivalent body will not be convened, as the study is approved by the ethical committees as a clinical trial with a CE-marked Medical Device with minimal risks. However, safety data will prospectively be reviewed at monthly TMC meetings.

The entered data will be verified by an independent unblinded study nurse, after a block of 10 subjects have been enrolled in the study.

### Audits and Inspections

A quality assurance audit/inspection of this study may be conducted by the Competent Authority or Competent Ethics Committee, respectively. The quality assurance auditor/inspector will have access to all medical records, the investigator’s study-related files and correspondence, and the informed consent documentation that is relevant to this clinical study. The investigator will allow the persons being responsible for the audit or the inspection to have access to the source data/documents and to answer any questions arising. All involved parties will keep the patient data strictly confidential.

The primary endpoint (MAL—14 AOU) and AEs will be monitored by the TMC.

### Anticipated Results

We expect that a 6-week program of wearing a wrist-worn activity tracking device, providing multimodal feedback on daily life arm use will induce statistically significant and clinical relevant changes in daily life upper limb use, when compared with wearing a sham device in patients who are beyond 3 months post-stroke.

## Discussion

By applying a wrist-worn activity tracking and feedback device, this study aims to optimize patient-reported amount of daily life upper limb use in stroke survivors beyond the first 3 months after stroke onset. Patients are eligible, regardless whether they have suffered an ischemic or hemorrhagic stroke. Although it has been shown that hemorrhagic stroke patients are more severely affected in the acute stage when compared with ischemic stroke patients (41), long-term functional outcomes were not significantly different (41, 42). As we include stroke survivors beyond 3 months post-stroke, we do not expect that type of stroke affects the outcomes of the present study. In addition, since we will recruit stroke patients beyond 3 months after stroke onset, we do expect that recovery has plateaued and no improvements will occur in the absence of intensive rehabilitation.

Previous studies have shown that intensive rehabilitation can influence upper limb capacity in chronic stroke patients, but context-specificity is often limited and the observed improvements fail to translate to upper limb use in daily life (10). The present study can help to close the gap that exists regarding the translation of gains made during inpatient rehabilitation to the home situation. The amount that patients use their paretic arm in daily life could be enhanced by providing multimodal feedback when they are in their home environment. Feedback can be used to motivate stroke patients and with that, positively impact patient outcomes (43). The experimental trial arm receives feedback regarding Arm Activities (i.e., knowledge of results). This can mainly be seen as extrinsic feedback, as the patients receive immediate vibrotactile feedback from the trackers when paretic arm use is below the pre-set daily target (i.e., controlled motivation). They furthermore receive delayed feedback regarding paretic arm use when they use the smartphone application. However, the use of the pre-installed application on the smartphone also requires self-determined motivation (i.e., self-control of feedback) of the patient, as he or she actively has to open the application for checking their progress and play the “Tree of Recovery.” We expect that the combination of various forms of feedback (vibrotactile feedback, visual feedback by the LED lights, information regarding upper limb activity counts in the app, and gamification) increases patient-reported amount of upper limb use in daily life, which consolidates at follow-up.

The applied tracking and feedback device focuses on the number of times that the patient moves the paretic upper limb in daily life. The MAL—14 AOU as a primary outcome matches this goal, by asking patients to rate how often they have used their paretic upper limb in daily life activities. We hypothesize that the change in MAL—14 AOU from baseline to post-intervention and follow-up will significantly differ between the experimental and control groups, favoring the experimental group. We expect that this difference will be also clinically relevant. This means that the change observed in the experimental group from baseline to post-intervention is at least 0.75 points more when compared with the control group. The latter is especially important, as clinical applicability and acceptability are highly dependent on whether a statistically significant difference is also meaningful for clinical practice. Although this study is not powered for our secondary outcomes, we expect to see relevant differences between groups regarding upper limb capacity (ARAT) and changes in upper limb activity counts as measured by the tracker devices itself during the 6-week intervention. Contrary, we do not expect to find clinically relevant change on motor function of the paretic upper limb (FMA—UE). First of all, because the feedback does not relate to QOM. Second, while patients in the chronic phase usually do not show significant, clinically relevant changes on motor function beyond 3 months post-stroke (44, 45). Furthermore, we do not expect to see a difference between groups regarding safety and compliance to wear the devices.

A positive result of this study will underline the importance of tracking and feedback on daily life upper limb use after stroke. It would provide therapists with a tool to enhance their patients’ real-world upper limb use, without being labor-intensive. Furthermore, the application of a tracking and feedback device could potentially reduce healthcare costs, although that is something that is beyond the scope of the present study.

## Ethics and Dissemination

This study will be carried out in compliance with the protocol approved by the previously mentioned ethical committees (version 1.1, dated 20/07/2017), the current version of the Declaration of Helsinki, the ICH-GCP or ISO 14155:2011, as well as all Swiss national legal and regulatory requirements. All subjects will give written informed consent in accordance with the Declaration of Helsinki. The trial is registered at https://clinicaltrials.gov, unique identifier NCT03294187. When protocol amendments are needed (e.g., to include another participating center), ethical approval will be obtained first. After having obtained this approval, relevant adaptations will be made in the relevant clinical trial registry databases.

Potentially eligible participants will be screened by the study site principal or sub-investigator for the presence of a first stroke >90 days, their ability to lift the paretic arm against gravity (>30° flexion or abduction), to don/doff the “ARYS™|tracker” devices independently on both wrists or if not, whether a caregiver is at hand for assistance, and to feel a soft touch on the dorsal side of their paretic wrist with closed eyes. Additionally, potential participants will be questioned and their medical record will be checked in regard to the other eligibility criteria. Given eligibility to take part in the study, they will be provided with further details and an informed consent form by one of the study members. The model consent form and other related documentation given to participants (all in German) can be obtained by the corresponding author upon request.

A separate list with patients screened, but who are not enrolled will contain information regarding the number of patients and the reasons for not enrolling. This list will be stored in the Trial Master File or Investigator Site File and is only accessible by the research team and the persons responsible for monitoring, audits, or inspections.

After the statistical analysis of this trial, the sponsor will make every effort to publish the data in a peer-reviewed medical journal, thereby adhering to the CONSORT reporting standards (46) and SPIRIT guidelines (47). The use of professional writers is not foreseen. Authorship eligibility is defined according to the International Committee of Medical Journal Editors. Furthermore, presentations at congresses and reporting in a patient and healthcare professional magazine are planned.

## Ethics Statement

The study is approved by the Cantonal Ethics Committees Zurich, and Northwest and Central Switzerland (BASEC-number 2017-00948) and registered in https://clinicaltrials.gov (NCT03294187) before recruitment started. This study will be carried out in compliance with the Declaration of Helsinki, ICH-GCP, ISO 14155:2011, and Swiss legal and regulatory requirements.

## Author Contributions

JH and JV participated in the experimental design. JH and JV were responsible for drafting, reviewing, and adapting the manuscript. AL was responsible for critically revising the manuscript. All authors approved the final draft of the manuscript.

## Conflict of Interest Statement

The sponsor AL is scientific advisor for Hocoma AG (Volketswil, Switzerland), which develops rehabilitation technology. However, there is no link between Hocoma and yband therapy AG. Furthermore, JH and AL have registered intellectual property (WO 2017/032765 A1) “Detecting an evaluating movements of a user.” All authors declare that the research will be conducted in the absence of any commercial or financial relationships that could be construed as a potential conflict of interest.
